# Assessment of satisfaction with antiretroviral drugs and the need for long-acting injectable medicines among people living with HIV in Japan and its associated factors: a prospective multicenter cross-sectional observational study

**DOI:** 10.1186/s12981-023-00557-5

**Published:** 2023-08-28

**Authors:** Masashi Ishihara, Shinichi Hikasa, Mariko Tsukiji, Yusuke Kunimoto, Kazuko Nobori, Takeshi Kimura, Kenta Onishi, Yuki Yamamoto, Kyohei Haruta, Yohei Kashiwabara, Kenji Fujii, Shota Shimabukuro, Daichi Watanabe, Hisashi Tsurumi, Akio Suzuki

**Affiliations:** 1 Department of Pharmacy, Gifu University Hospital, 1-1, Yanagido, Gifu, 501-1194 Japan; 2 Department of Pharmacy, Hyogo Medical University Hospital, Nishinomiya, Hyogo Japan; 3 Division of Pharmacy, Chiba University Hospital, Chiba, Chiba Japan; 4 Department of Hospital Pharmacy, Sapporo Medical University Hospital, Sapporo, Hokkaido Japan; 5 Department of Pharmacy, Kobe City Medical Center General Hospital, Kobe, Hyogo Japan; 6 Department of Pharmacy, Kobe University Hospital, Kobe, Japan; 7 Division of Pharmacy, Wakayama Medical University Hospital, Wakayama, Wakayama Japan; 8 Division of Pharmacy, Himeji Medical Center, Himeji, Hyogo Japan; 9 Department of Pharmacy, Nara Medical University Hospital, Kashihara, Nara Japan; 10 Division of Pharmacy, Japanese Red Cross Kyoto Daiichi Hospital, Higashiyama, Kyoto Japan; 11 Department of Pharmaceutical Services, Hiroshima University Hospital, Hiroshima, Hiroshima Japan; 12 Department of Haematology and Infectious Disease, Gifu University Hospital, Yanagido, Gifu Japan; 13 Laboratory of Advanced Medical Pharmacy, Gifu Pharmaceutical University, Daigakunishi, Gifu Japan

**Keywords:** Antiretroviral therapy, Patient satisfaction, People living with HIV, Long-acting injectable

## Abstract

**Background:**

Long-acting injectable formulations for HIV infection have been approved and are now available in Japan. Although not currently recommended as first-line drugs in Japanese or overseas guidelines, use of such formulations may increase, in accordance with patient conditions and preference. We determine the level of satisfaction with current anti-HIV drugs and analyzed the preferences of patients who favor long-acting injectable drugs based on their satisfaction level with the present anti-HIV drugs.

**Methods:**

People living with HIV (PLWH) who had received antiretroviral therapy (ART) for at least one month and consented to the study between 1 April and 31 December 2021 were included in a survey conducted using a self-administered questionnaire. The content of the survey included satisfaction with seven items (tablet size, ease and feeling when taking the medicine, color, taste, portability, daily oral therapy, and co-payment) related to the anti-HIV drugs they were taking and their need for future drugs (dosage form, frequency of dosing, long-acting injectable, etc.). In addition, factors related to the need for long-acting injectable medications were analyzed with regard to the relationship with satisfaction with anti-HIV drugs.

**Results:**

Overall, 667 patients available for analysis were included in this study. Satisfaction with anti-HIV drugs was highest with regard to “co-payment” and lowest with “daily oral therapy”. Regarding the need for long-acting injectable medications, logistic regression analysis indicated that tablet size and daily oral therapy were significant predictors of patient preference for a once-every-eight-weeks intramuscular formulation in terms of their requirement for long-acting injectable medications (tablet size, OR = 2.14, 95%CI 1.030–4.430, p = 0.042; and daily oral therapy, OR = 1.75, 95%CI 1.010–3.030, p = 0.044).

**Conclusions:**

Patients currently receiving anti-HIV drugs who express dissatisfaction with tablet size and daily oral therapy may prefer a long-acting injectable formulation, taking into consideration patient age, employment status, ART history, frequency of daily dosage and concomitant medications other than ART.

## Background

Advances in the efficacy of antiretroviral therapy (ART) for HIV infection have enabled many HIV-infected people to maintain virologic suppression. Current ART has significantly reduced mortality and improved the quality of life of people living with HIV (PLWH) [1–3], a benefit considered attributable to the development of drugs with strong antiviral activity and the appearance of combination drugs containing two or three ingredients in a single tablet regimen (STR). In Japan, it is reported that more than 90% of patients receiving ART have achieved virological suppression [4][5]. Nevertheless, it is now necessary to continue ART for life because anti-HIV drugs do not yet have the capacity to completely eliminate HIV from the body, and patients are consequently required to continue anti-HIV medication for almost their entire lives [6]. Since patient adherence to ART is essential to avoiding drug resistance and virologic failure during long-term treatment, a better understanding of satisfaction with patient treatment may help promote ongoing medication adherence. Since PLWH must remain on ART for a long period of time, there is concern that they may tire of taking the medicine and as a result decrease adherence. Accordingly, treatment of HIV infection requires more than just virological suppression with anti-HIV drugs - attention must also be given to improving quality of life by reducing side effects and improving the convenience of oral administration [7].

Recently, new long-acting injectable formulations for HIV infection have been approved and are now available in Japan. Although not currently recommended as first-line drugs in Japanese [8] or overseas guidelines [9–11], use of such formulations in Japan is predicted to increase, in accordance with patient conditions and preference. Factors encouraging use include the lengthening of PLWH survival [12, 13], aging of PLWH [14], and the possibility that some patients may not be able to take drugs orally in the future due to problems such as dysphagia. Additionally, concern has been expressed that the long-term success of treatment may be affected by the emotional and adherence issues associated with daily ART. However, it is currently unclear whether the new long-acting injectable formulations should be recommended for all patients, or for which patient groups. In particular, PLWH in Japan were not surveyed regarding which anti-HIV drug formulation they preferred before the long-acting injection drugs were approved.

Here, to encourage more appropriate medication selection for patients, we aimed to discern patient satisfaction with current antiretroviral therapy and the need for long-acting injectable medications. We also examined the correlation between patient need for long-acting injectable drugs and factors that contribute to their satisfaction.

## Methods

### Study design and participants

We conducted an anonymous, multi-center, prospective, cross-sectional paper‐based survey involving PLWH on ART who visited the outpatient clinic between 1st April 2021 and 31st December 2021. Inclusion criteria were (a) current receipt of ART for over 4 weeks, (b) age 20 years or older, and (c) reading and writing ability in Japanese language. There were no exclusion criteria.

A multi-center hospital recruited potential participants across Japan and distributed questionnaires to them. Informed consent for enrollment was obtained in writing by checking of a consent box on the questionnaire document. Responses to the questionnaire were voluntary, and confidentiality was maintained throughout all investigations and analyses. The ethical validity of the study was approved by the institutional review board of Gifu University Hospital (no. 2021-027), the main institution, and all the other study sites.

### Measurements

#### Patient satisfaction with medications for anti-HIV drugs

Satisfaction was assessed using an independently developed satisfaction questionnaire for anti-HIV drugs. The questionnaire included 7 items phrased in various ways related to taking anti-HIV drugs (tablet size, ease and feeling when taking the medicine, color, taste, portability, daily oral therapy, and co-payment) (Table 1). These phrases were developed with particular care and discussion between the researcher and an HIV infection specialist pharmacist regarding the content, clarity, and format of the items.

**Table 1:** Questionnaire items for patient satisfaction with anti-HIV medications

	Item label	Item wording	
1	Tablet size	How satisfied are you with the size of the anti-HIV drugs you are taking?	
2	Ease and feeling when taking the medicine	How satisfied are you with the ease and feeling of taking the anti-HIV drugs you are taking?	
3	Color	How satisfied are you with the color of the anti-HIV drugs you are taking?	
4	Taste	How satisfied are you with the taste of the anti-HIV drugs you are taking?	
5	Portability	How satisfied are you with the portability of the anti-HIV drugs you are taking?	
6	Daily oral therapy	How satisfied are you with the daily oral therapy of the anti-HIV drugs you are taking?	
7	Co-payment	How satisfied are you with the co-payment of the anti-HIV drugs you are taking?	

The questionnaire included seven items related to anti-HIV drug taking (tablet size, ease and feeling when taking the medicine, color, taste, portability, daily oral therapy, and co-payment). Participants ranked each item on satisfaction with anti-HIV drugs on a 7-point Likert scale ranging from 6 (“very satisfied”) to 0 (“not at all satisfied”)

Participants were asked, “How satisfied are you with the anti-HIV medications you are currently taking?“ and responded by selecting the response that best reflected what type of antiretroviral drug they would like to use for their treatment needs (scored on a 7-point Likert scale, with anchors: 0 = not at all satisfied, 1 = not satisfied, 2 = somewhat unsatisfied, 3 = neither satisfied not dissatisfied, 4 = somewhat satisfied, 5 = satisfied, 6 = very satisfied).

#### Needs of PLWH taking anti-HIV drugs for future anti-HIV drug formulation of a drug dose

Needs were assessed using an independently developed questionnaire regarding future anti-HIV agents (dosage form, frequency of dosing, long-acting injectable, etc.). The survey consists of 15 patterns of options pertaining to administration frequency, dosage form, and frequency of visits to hospital, including prospective forms that may become available in the future (Table 2). These options were developed by discussion between the researcher and a specialist pharmacist in HIV infection regarding their content. Participants were asked, “What type of anti-HIV drugs would you want to use based on your experience?“ They were solicited to elect the most suitable anti-HIV drugs for their individual treatment needs by selecting the formulation type that most optimally aligned with their needs from a list of available options in multiple-choice format.

**Table 2:** Options regarding the formulation of future anti-HIV drugs that meet patient needs

	Formulation of drug	Frequency of dosage	Frequency of visit to hospital	
1	Tablet	Every day	Every 3 months	
2	Tablet	Once a week	Every 3 months	
3	Gummies	Every day	Every 3 months	
4	Jelly	Every day	Every 3 months	
5	Lozenges	Every day	Every 3 months	
6	OD tablet	Once a week	Every 3 months	
7	OD tablet	Every day	Every 3 months	
8	S.C.	Once a month	Every 3 months	
9	S.C.	Every 3 months	Every 3 months	
10	I.M.	Every 4 weeks	Every month	
11	I.M.	Every 8 weeks	Every 2 months	
12	Patches	Every day	Every 3 months	
13	Patches	Every 2-3days	Every 3 months	
14	Implant	Every 6 months	Every 3 months	
15	Implant	Every year	Every 3 months	

OD: Orally disintegrating tablet, S.C: subcutaneous injection, I.M: intramuscular injection, Implant: implantable DDS (potential future drug formulations)

#### Data collection

Patient information was collected from electronic medical records, including age, sex, ART regimen, and duration of treatment with overall ART and of concomitant medications; and from the survey, including satisfaction with anti-HIV drugs, employment status and preference for future anti-HIV drugs etc. Satisfaction-related self-administered questionnaires were collected from the patient by hand delivery or postal mail.

### Statistical analyses

The distribution of continuous variables was determined using the Kolmogorov–Smirnov normality test. Continuous data with a normal distribution and abnormal distribution are shown as mean ± standard deviation (SD) and median (interquartile range [IQR]) values, respectively. Satisfaction rates were calculated in descriptive analysis to characterize the range of issues related to PLWH satisfaction with anti-HIV drugs in relation to daily oral ART. Formulation of anti-HIV drugs was also calculated in descriptive analysis to characterize the range of needs related to anti-HIV drugs that PLWH prefer in the future. Satisfaction with current anti-HIV drugs taken by people living with HIV (PLWH) was compared between those who expressed a preference to receive an intramuscular injection every 8 weeks and those who did not. Student’s t-test was used for normally distributed continuous variables, the Mann-Whitney U test was applied for nonparametric continuous variables, and Fisher’s exact test was utilized for categorical variables.　A logistic regression model was employed to examine factors associated with patient satisfaction for those who expressed a preference for 8-week intramuscular injection, utilizing the questionnaire items (tablet size, ease and feeling when taking the medicine, color, taste, portability, daily oral therapy, and co-payment) as comparative variables. In the logistic regression model, covariates such as patient age, employment status, duration of receipt of overall ART (medication history), frequency of daily dosage, and daily tablet dosage (including concomitant medications other than ART) were included using a forced entry method. These factors were selected based on their potential to influence the preference for 8-week intramuscular injections. In all statistical evaluations, results were considered significant at p < 0.05. All analyses were performed using R version 4.2.2 (The R Project for Statistical Computing).

## Results

### Study population characteristics

A total of 735 patients from 11 hospitals responded to the questionnaire. Of these, 68 were excluded because of the lack of written consent or an incomplete response to questionnaires. Finally, we included 667 patients. Table 3 summarizes the baseline clinical characteristics of individuals.

**Table 3:** Characteristics of participants

Age, median (IQR)-yr	47 (40–55)
Age group, n (%)	
<50 years	394 (59)
≥50 years	273 (41)
Sex, n (%)	
Male	635 (95)
Female	32 (5)
CD4 cell counts, median (IQR)-cells/µL	587 (444–757)
HIV-RNA, n (%)	
≤ 20 copies/mL	548 (82)
20‒49 copies/mL	52 (15)
≥ 50 copies/mL	20 (3)
ART regimen consisting of the following tablet sizes, n (%)	
Tablets size less than 15 mm	342 (51)
Tablets size over 15 mm	325 (49)
Duration of receipt of overall ART (medication history), n (%)	
≤ 3 years	95 (14)
≥ 3 years	572 (86)
Employment, n (%)	
Employed	530 (79)
Unemployed	110 (16)
Unknown	27 (4)
Frequency of daily dosage, n (%)	
Once a day	392 (59)
Twice a day	128 (19)
Three times a day	82 (12)
4 or more times a day	65 (10)
Concomitant medications, n (%)	
With concomitant medications	379 (57)
Without concomitant medications	285 (43)
Unknown	3
Daily tablet dosage (including concomitant medications), n (%)	
1 tablet/day	193 (29)
2 tablets/day	139 (21)
3 tablets/day	99 (15)
4 or more tablets/day	236 (35)

IQR: interquartile range, AIDS: acquired immune deficiency syndrome, ART: antiretroviral therapy

### Satisfaction with each item for anti-HIV drugs

**Fig. 1:**
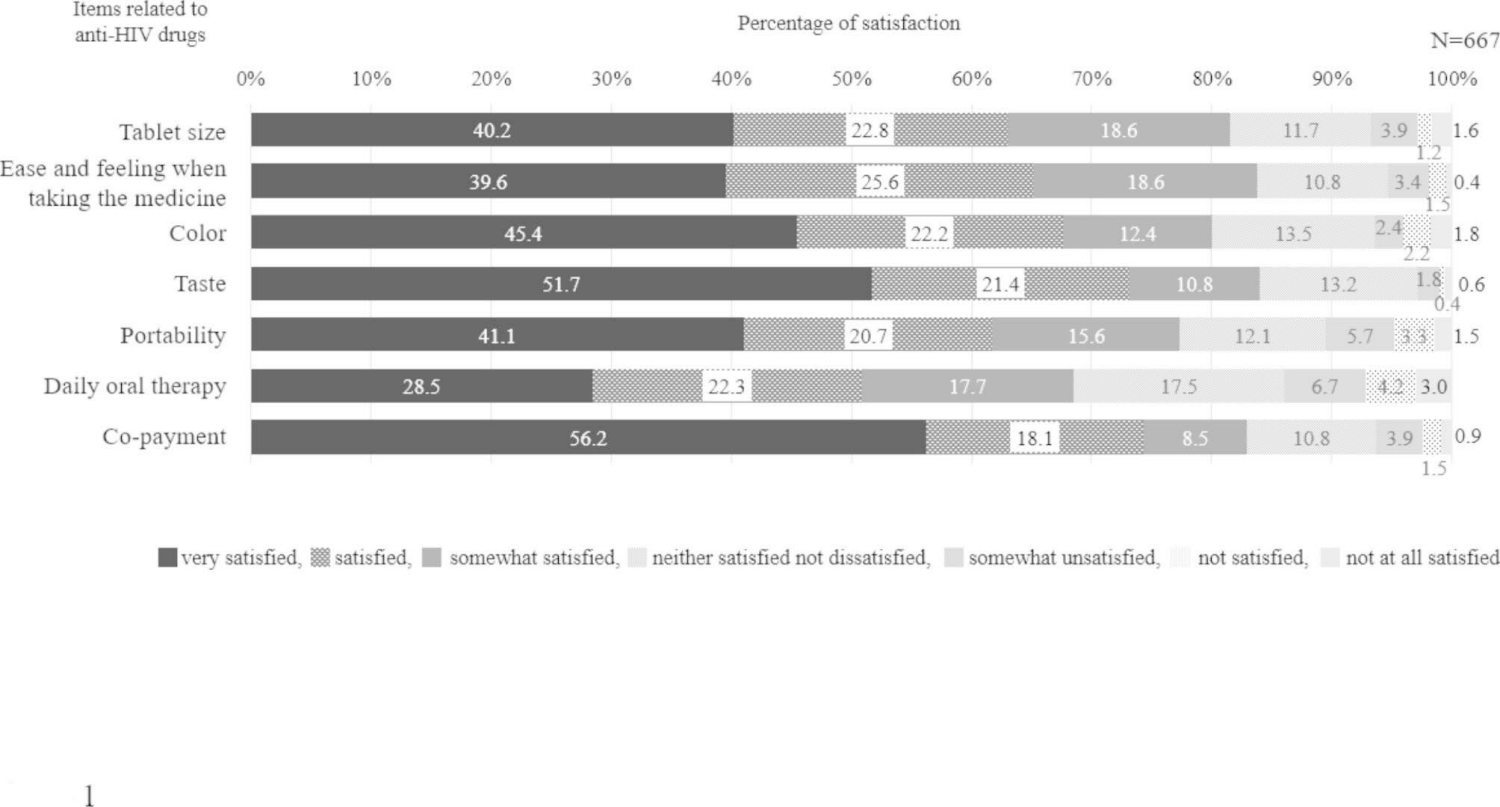
Satisfaction with individual items among anti-HIV drugs. The questionnaire included seven items related to anti-HIV drug taking (tablet size, ease and feeling when taking the medicine, color, taste, portability, daily oral therapy, and co-payment). Participants ranked their answers to each item on a 7-point Likert scale, ranging from 6 (“very satisfied”) to 0 (“very dissatisfied”)

Figure 1 shows satisfaction with the current anti-HIV drugs. The item with the highest percentage of “very satisfied” was “co-payment” (n = 286, 56.2%), while “daily oral therapy” (n = 190, 28.5%) had the lowest.

### PLWH needs for preferred anti-HIV drugs

**Fig. 2:**
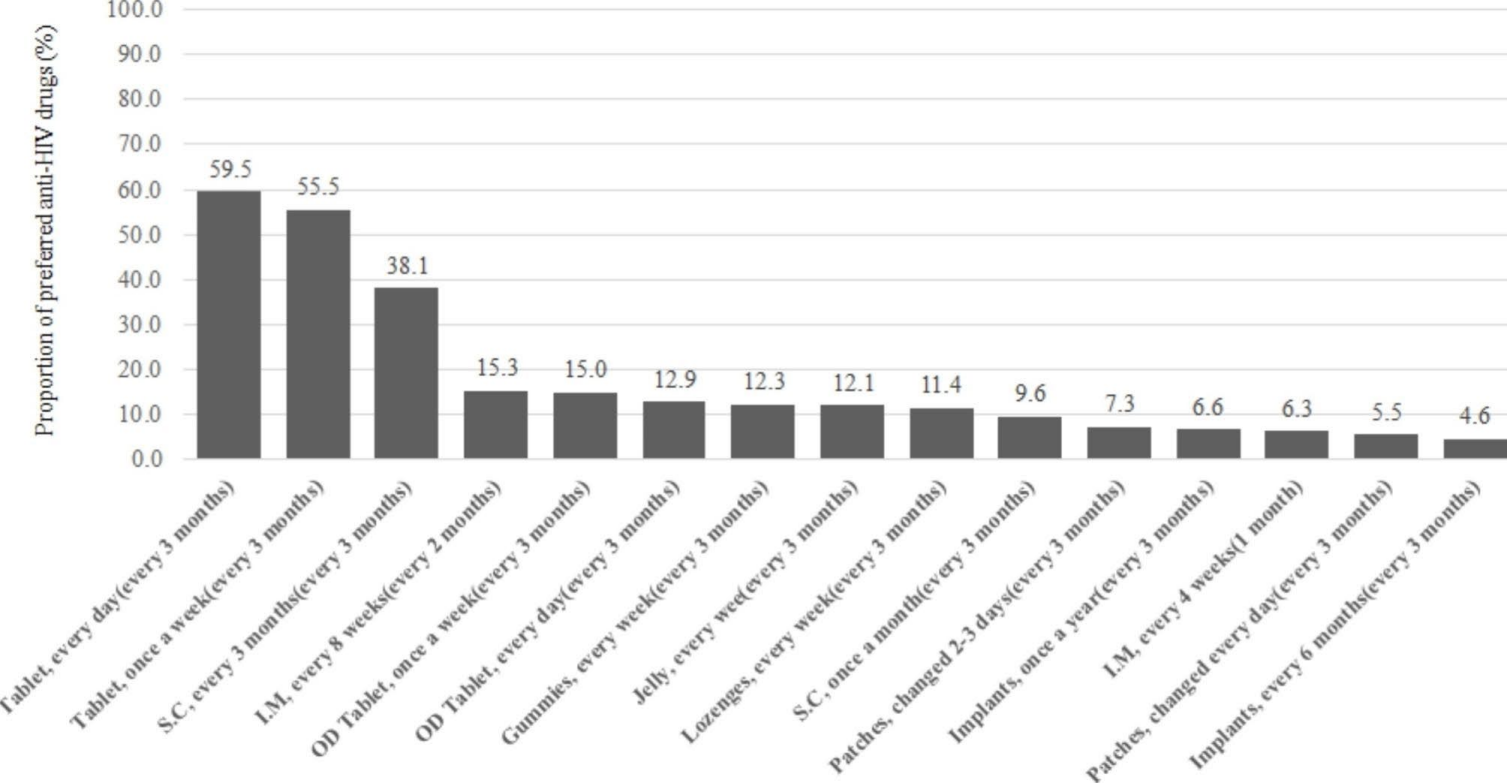
PLWH needs for preferred anti-HIV drugs in the future. The questionnaire was designed and conducted to allow multiple answers from a list of options regarding new or other formulations of anti-HIV drugs that PLWH would like to receive. Abbreviations and parentheses are as follows. Each item indicates the formulation of a drug and its frequency of administration, (frequency of visit to hospital). Tab: tablets, S.C: subcutaneous injection, I.M: intramuscular injection, OD: orally disintegrating tablets

Figure 2 shows PLWH needs for preferred anti-HIV drugs (dosage form, frequency of dosing, etc.) for drug doses in the future. This question allowed multiple responses. In terms of therapeutic drug needs, the most prevalent demands from patients were for “daily oral tablets (with a visit frequency of once every 3 months)” (n = 397, 59.5%), followed by “weekly oral tablets (with a visit frequency of once every 3 months)” (n = 370, 55.5%), “subcutaneous injections, once every 3 months (with a visit frequency of once every 3 months)” (n = 254, 38.1%), and “intramuscular injections once every 8 weeks (with a visit frequency of once every 2 months)” (n = 102, 15.3%).

### Relationship between need for long-acting injectable (every 8 weeks) and satisfaction with current anti-HIV drugs

Next, we examined the relationship between the need for a long-acting injectable formulation (every 8 weeks) and satisfaction with current anti-HIV drugs. Table 4 shows characteristics of patients with and without “prefer long-acting drugs (every 8 weeks)”.

**Table 4:** Comparison of participants with and without preference for long-acting drugs administered every 8 weeks

	With preference for long-acting drugs	Without preference for long-acting drugs	P value
Age, median (IQR)-yr	45 (39–50)	48 (40–57)	0.007^a)^
Age group, n (%)			0.001^b)^
< 50 years	75 (4)	319 (5)	
≥ 50 years	27 (96)	246 (95)	
Sex, n (%)			0.805^b)^
Male	98 (96)	537 (95)	
Female	4 (4)	28 (5)	
CD4 cell counts, median (IQR)-cells/µL	615 (475–755)	580 (438–757)	0.315^a)^
HIV-RNA, n (%)			1.000^b)^
≤ 20 copies/mL	91 (89)	504 (89)	
20-49 copies/mL	8 (8)	44 (8)	
≥ 50 copies/mL	3 (3)	17 (3)	
ART regimen consisting of the following tablet sizes, n (%)			1.000^b)^
Tablets size less than 15 mm	52 (51)	291 (51)	
Tablets size over 15 mm	50 (49)	274 (49)	
Duration of receipt of overall ART (medication history), n (%)			0.122^b)^
< 3 years	20 (20)	75 (13)	
≥ 3 years	82 (80)	490 (83)	
Employment, n (%)			0.469^b)^
Employed	86 (86)	444 (79)	
Unemployed	14 (14)	93 (16)	
Unknown	2 (2)	28 (5)	
Frequency of daily dosage, n (%)			0.908^b)^
Once a day	58 (57)	334 (59)	
Twice a day	19 (19)	109 (19)	
Three times a day	14 (14)	68 (12)	
4 or more times a day	11(10)	54 (10)	
Concomitant medications^*^, n (%)			0.913^b)^
With concomitant medications	57 (56)	322 (57)	
Without concomitant medications	44 (43)	241 (43)	
Unknown	1	2	
Daily tablet dosage^†^, n (%)			0.851^b)^
1 tablet/day	26 (25)	167 (30)	
2 tablets/day	22 (22)	117 (21)	
3 tablets/day	15 (15)	84 (15)	
4 or more tablets/day	39 (38)	197 (35)	

Data are expressed as the number (percentage) or median (IQR). AIDS, acquired immune deficiency syndrome; ART, antiretroviral therapy

^*^Concomitant medications other than ART, ^†^Daily Tablet Dosage; Number of tablets taken daily including concomitant medications other than ART

^(a)^ Mann-Whitney U test; ^(b)^ Fisher’s exact test

**Fig. 3:**
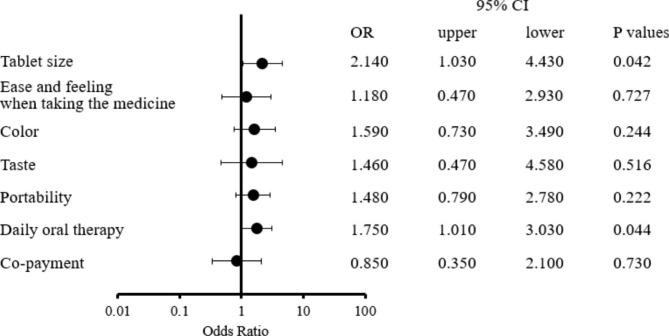
Logistic regression model incorporating the questionnaire items (tablet size, ease and feeling when taking the medicine, color, taste, portability, daily oral therapy, co-payment) as comparative factors. Outcomes are characterized as patient propensity for an 8-week injectable formulation. The estimated likelihood of this preference was deduced by considering not only the questionnaire items but also the confounders of patient age, employment status, duration of receiving overall ART (medication history), frequency of daily dosages and daily tablet dosage (including concomitant medications other than ART).

A logistic regression analysis which utilized the outcome of patients requesting 8-week long-acting injectable medication and employed questionnaire items (including tablet size, ease and feeling when taking the medicine, color, taste, portability, daily oral therapy, and co-payment) as comparative variables showed that “tablet size” and “daily oral therapy” were statistically significant factors (tablet size, OR = 2.14, 95%CI 1.030–4.430, p = 0.042; and daily oral therapy, OR = 1.75, 95%CI 1.010–3.030, p = 0.044) (Fig. 3).

## Discussion

In this study, we investigated patient satisfaction with current anti-HIV drugs, and surveyed patient needs for long-acting injectable medications prior to approval of these drugs in Japan to determine for which patients long-acting injectable medications would be appropriate. The results showed that although patients were generally satisfied with most items used to determined satisfaction with anti-HIV drugs, satisfaction with “daily oral therapy” was lowest. In addition, we also found that there is a need for long-acting injectable drugs in Japan, which may be suitable for patients who are dissatisfied with the tablet size and daily oral therapy of current anti-HIV drugs.

Current guidelines for HIV infection recommend ART in the form of one tablet once daily as primary treatment [8, 9]. Currently recommended anti-HIV drugs have stronger antiviral activity and higher genetic barriers than previous anti-HIV drugs. It has been reported that once-daily, single-tablet regimen (STR) initiators were significantly less likely to discontinue therapy and had greater adherence and persistence compared to multi-tablet regimen (MTR) initiators among patients newly prescribed ART [15]. Accordingly, reducing the frequency of dosing and the number of pills taken per day is thought to have alleviated the burden on patients. Nevertheless, it is reported that 38.7% of patients would like to see the advent of long-acting drugs that do not require daily dosing as a priority improvement in the treatment of HIV infection [16]. QD (once daily) is the most convenient way to take ART, as recommended by current practice guidelines, but even with QD, it is reported that once-daily administration can be emotionally taxing for patients because it triggers patients to remind themselves that ‘I have HIV infection’ [17]. As reported elsewhere by Koga et al [16], the results of this study were inferred to mean that satisfaction with anti-HIV drugs is affected by the burden of daily oral therapy. Regarding patient needs for anti-HIV drugs, the results showed that most patients preferred the following formulation types among the many options available: “daily or weekly tablets,“ “once every three months subcutaneous formulation,“ and “once every eight weeks intramuscular formulation”. We infer from the relatively high preference for weekly tablets and two- or three-monthly injectable formulations that many patients feel burdened by daily oral medication, whereas other patients are satisfied with their current once-daily medication.

Recently, long-acting injectable drugs have been approved in Japan, and many hospitals have begun to use them. We conducted the present survey before the approval of these drugs and simultaneously investigated patient need for long-acting injectable drugs (Q8W). The findings indicate that patients who preferred the once-every-eight-week intramuscular formulation (Q8W) were dissatisfied with “tablet size” or “daily oral therapy” of their current anti-HIV drugs.

Pharmaceutical tablets typically range in size from 5 to 10 mm, but anti-HIV drugs tend to be larger. The smallest tablet size for anti-HIV medication is 6.4 mm (EDURANT® Tablets), while the largest is 23 mm (PREZCOBIX® Combination Tablets). We therefore assumed that patients were dissatisfied with tablet sizes and wished for an injectable form.

In many PLWH treated with anti-HIV medications, their HIV infection is controlled by ART and they are able to lead a daily life similar to that of non-HIV-infected people. In addition, many PLWH have improved symptoms and return to society following treatment and are in general employment and have a variety of social backgrounds. It is therefore considered important to choose a drug that fits the patient’s lifestyle to ensure adherence in perpetuity. It has also been reported that Shared Decision Making with the patient is an important communication strategy when initiating or changing ART to select a more appropriate drug for the patient [18, 19].

In the present investigation, it is possible that as PLWH underwent prolonged ART, they may have opted for a once per eight weeks injectable drug due to the inadequacy of their current anti-HIV drugs in accommodating their lifestyle, and that dissatisfaction with “daily oral therapy” was one contributing factor of the reasons why they chose long-acting injectable drugs in this preference.

In general, comorbidities and the number of medications increase in older age. It is similarly reported that HIV-infected patients have more comorbidities and medications than non-infected patients [20]. Polypharmacy in those aged over 50 years old has also been reported [21, 22]. Nevertheless, Hinkin et al. reported that mean ART adherence rate was significantly greater in patients ≥ 50 years of age than in younger patients, and that HIV-infected persons ≥ 50 years of age were three times more likely than their younger counterparts to achieve adherence rates of 95% or greater [23].

The present results suggest that a lower proportion of patients aged over 50 years may have requested the Q8W formulation because they are more likely to be taking concomitant medications other than anti-HIV drugs, and that this daily administration requirement would remain if their anti-HIV drugs but not their other drugs were switched to injectable drugs. We also speculated that whereas patients with longer medication histories were accustomed to taking anti-HIV drugs, those with shorter medication histories were not accustomed to daily medication, and accordingly may have wished that the burden of daily medication for a medicine required for the rest of their lives could be eliminated.

### Strengths and limitations

The strength of our study is that, because it was conducted to investigate patients’ needs for anti-HIV drugs before the approval of long-acting injection drugs in Japan, we were able to obtain their honest thoughts. Nonetheless, limitations also exist. First, we can only infer associations because of the cross-sectional design. Second, the findings may have limited generalizability because participants were limited to PLWH with reading and writing ability in Japanese, and only a limited number of countries were included. Third, the PLWH Satisfaction and Needs for Anti-HIV Drugs Questionnaire was developed by discussion between the researcher and an HIV infection specialist pharmacist regarding the content, so it is possible that satisfaction with anti-HIV drugs was not perfectly assessed. Despite these limitations, this study offers the opinion of anti-HIV drugs held by PLWH, which can serve as a reference for future anti-HIV drug development and in the selection of new anti-HIV drug formulations sought by patients.

## Conclusion

This study showed that daily oral therapy affects satisfaction with anti-HIV drugs, even though regimens use the simplest once-daily single tablet regimen. This study suggests that long-acting injection drugs may better meet the needs of patients who are not satisfied with tablet size and daily oral therapy of current drugs, with suitable consideration to patient age, employment status, ART history, frequency of daily dosages and the presence or absence of concomitant medications other than ART.

## Data Availability

The materials, datasets used and analyzed in the current study are available from the corresponding author upon reasonable request.

## Abbreviations

PLWH: people living with HIV
ART: Antiretroviral Therapy
STR: Single Tablet Regimen
MTR: Multi-Tablet Regimen
QD: Quaque Die(once daily)
Q8W: Quaque 8 weeks(8-weekly)
OD: Orally disintegrating tablet
S.C.: subcutaneous injection
I.M.: intramuscular injection
Implant: Implantable Drug Delivery System
